# NPM1 histone chaperone is upregulated in glioblastoma to promote cell survival and maintain nucleolar shape

**DOI:** 10.1038/srep16495

**Published:** 2015-11-12

**Authors:** Karl Holmberg Olausson, Tamador Elsir, Kaveh Moazemi Goudarzi, Monica Nistér, Mikael S. Lindström

**Affiliations:** 1 Department of Oncology-Pathology, Karolinska Institutet, Cancer Center Karolinska, CCK R8:05, Karolinska University Hospital in Solna, SE-17176 Stockholm, Sweden; 2 Department of Neuroscience, Neurology, Uppsala University, Uppsala University Hospital, SE-75185 Uppsala; 3 Science for Life Laboratory, Division of Translational Medicine and Chemical Biology, Department of Medical Biochemistry and Biophysics, Karolinska Institutet, SE-171 21, Stockholm, Sweden

## Abstract

Glioblastoma (grade IV glioma) is the most common and aggressive adult brain tumor. A better understanding of the biology of glioblastoma cells is crucial to identify molecular targets stimulating cell death. NPM1 (nucleophosmin) is a multifunctional chaperone that plays an important role in cancer development. Herein, NPM1 was analyzed by immunohistochemistry in human astrocytic gliomas. NPM1 was detected in all tumors but with a significantly higher staining intensity in grade IV than in low grade tumors. Depletion of NPM1 had only modest effects on the viability of U251MG, U1242MG, and U343MGa Cl2:6 glioma cells, despite alterations in nucleolar morphology. Glioma cell cultures depleted of NPM1 exposed to micromolar levels of actinomycin D were more prone to cell death (apoptosis) compared to cultures retaining NPM1. We had previously found that NPM1 binds to linker histone H1.5. Here we could show that silencing of H1.5 triggered glioma cell apoptosis as evidenced by a marked increase in both the numbers of cleaved caspase-3^+^ cells and in the amounts of cleaved PARP. Enforced expression of NPM1 suppressed apoptosis in H1.5 depleted glioma cells. Although our studies would suggest little effectiveness of targeting NPM1 alone there could be potential using it as a combination treatment.

Glioblastoma is the most common and aggressive primary brain tumor in adults. Treatment of glioblastoma is difficult and can extend patients’ lives by only a few months^1^. Nevertheless, survival for patients with glioblastoma has improved over the past decade from an average of 10 months to 14 months after diagnosis due to improvements in the standard treatments^1^. Gliomas are thought to arise from neural stem cells, glial progenitor cells or even from de-differentiated astrocytes^2^. Astrocytic gliomas are classified by the WHO into four grades: grade I pilocytic astrocytoma, grade I subependymal giant cell astrocytoma, grade II pleomorphic xanthoastrocytoma, grade II astrocytoma (low grade-diffuse), grade III anaplastic astrocytoma, and grade IV glioblastoma^3^. The glioblastomas are diffusely infiltrating, poorly differentiated tumors, with a high degree of cellular polymorphism, high proliferative activity, necrosis and extensive micro-vascularization^4^. In glioblastoma cells, several mechanisms responsible for induction of apoptosis are blocked, while chaperones promoting cell survival are overexpressed^5^,^6^,^7^. The chaperone NPM1 functions in diverse cellular processes including centrosome duplication, ribosome biogenesis, intracellular transport, chromatin remodeling (core and linker histone binding), apoptosis, and mRNA splicing^8^,^9^. Elevated levels of NPM1 protein have been detected in cancers of the stomach^10^, breast^11^, colon^12^, bladder^13^, prostate^14^, and the thyroid^15^. It has also been found to be overexpressed in gliomas at both mRNA and protein levels when compared to normal brain^16^,^17^,^18^,^19^. Moreover, chromosomal translocations involving *NPM1* occur in several types of leukemia and lymphoma^9^, and one-third of adult acute myeloid leukemia cases display aberrant cytoplasmic expression of NPM1 due to mutations occurring in the 12^th^ exon^20^. NPM1 has been ascribed both growth promoting and tumor suppressive functions^9^,^21^. For example, its overexpression transforms immortalized NIH3T3 cells, blunts the activation of p53 by the ARF tumor suppressor, and facilitates DNA replication and DNA repair^22^,^23^. In contrast, loss of NPM1 destabilizes ARF and also weakens the p53 response^24^. Loss of NPM1 results in genome instability manifesting itself with aneuploidy, increase in centrosome numbers, and DNA damage checkpoint activation^23^,^25^,^26^. NPM1 may play a protective role against oxidative stress in hematopoietic stem cells^9^. Several different types of cancer cells with elevated levels of NPM1 are also more resistant to UV or hypoxia induced apoptosis than those with low expression^27^. Such anti-apoptotic functions have been connected with NPM1´s ability to prevent p53’s localization to mitochondria^28^. Also, by preventing BAX mitochondrial translocation and activation, NPM1 helps liver carcinoma cells to evade apoptosis in a p53-independent manner^29^. Npm1 is an essential protein for normal development and knockout mice display aberrant organogenesis resulting in death of the mice between embryonic day E11.5 and E16.5 due to anemia^30^. However, Npm1 is also required for the proper development of the forebrain in mice^30^, and the Npm1 deficient embryos lack proper forebrain with the subdivision between metencephalon and mesencephalon shifted anteriorly. Analysis of neural tissues revealed marked apoptosis suggesting a crucial function of Npm1 in normal brain development^30^.

## Results

### High levels of NPM1 in glioblastoma

**Figure 1:**
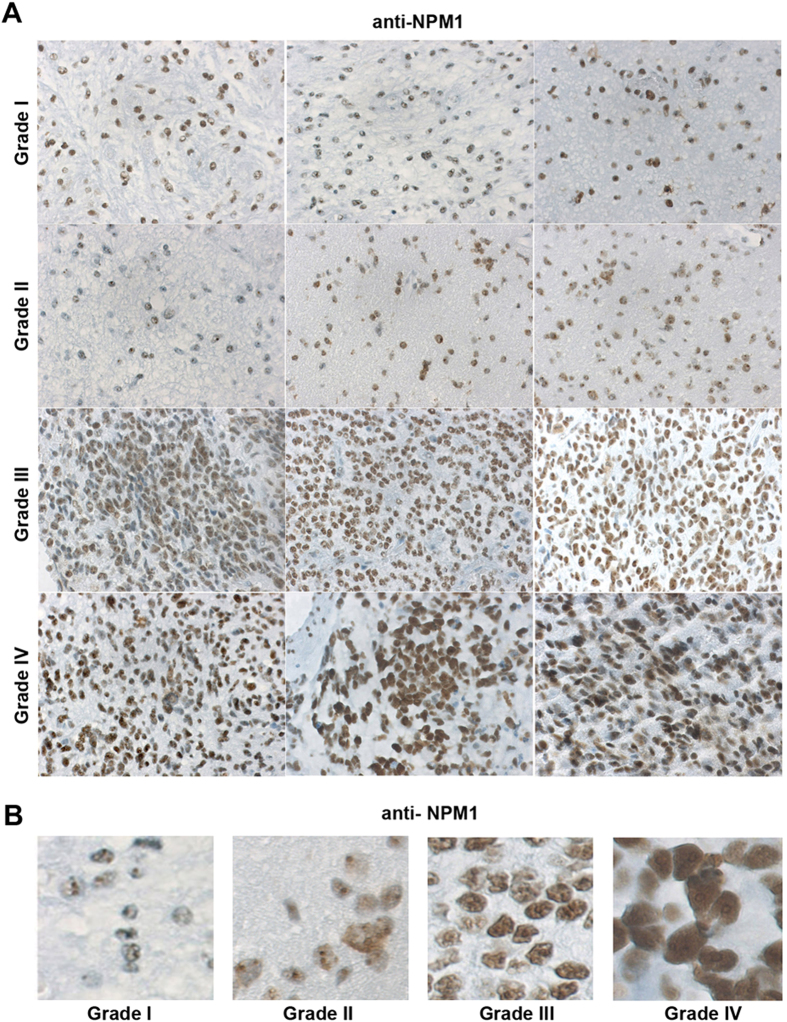
Detection of NPM1 in astrocytic gliomas. (**A**) Immunohistochemical staining of NPM1 (brown) in astrocytic glioma tumors of grades I, II, III and IV. Three tumor samples from different patients are shown for each grade. Obj. 20x. (**B**) Representative zoom-in micrographs showing NPM1 staining in tumor samples of different grades (I-IV).

**Figure 2:**
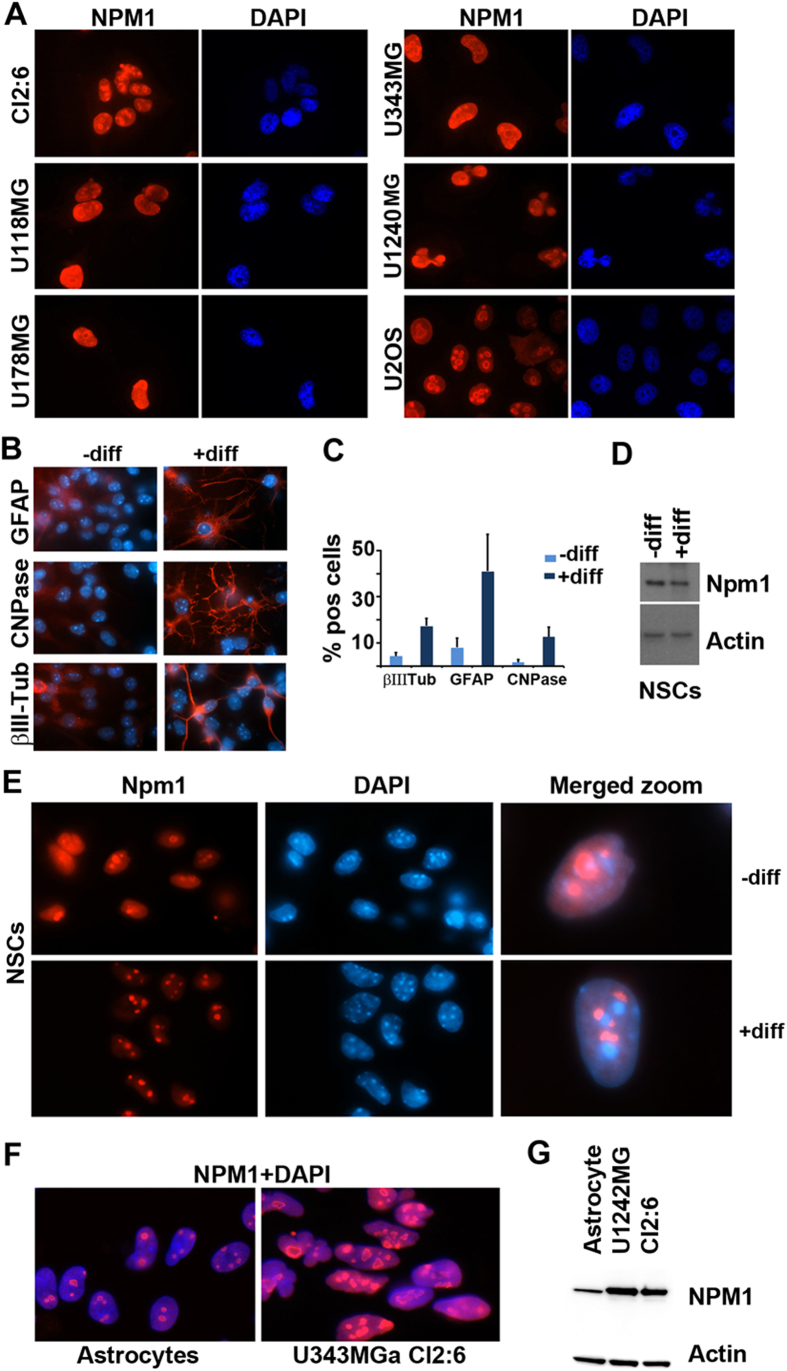
Levels and localization of NPM1 in human glioma cell lines and mouse neural stem cells. (**A**) IF staining of NPM1 (red) in glioma cell lines as indicated in the figure. Nuclei were counterstained with DAPI (blue). U2OS osteosarcoma cells were used in comparison. (**B**) IF staining (red) of GFAP (astrocyte marker), CNPase (oligodendrocyte marker) and βIII-tubulin (neuronal marker) in either undifferentiated (–diff) or differentiated (+diff) mouse wild type NSCs. Nuclei were counterstained with DAPI (blue) and shown merged with the red signal. In this experiment, mouse NSCs were growing as spheres and thereafter dissociated onto laminin coated coverslips and induced to differentiate or maintained in an undifferentiated state. (**C**) Percentages of GFAP^+^, CNPase^+^, and βIII-tubulin^+^ cells in undifferentiated (–diff) or differentiated (+diff) mouse NSCs. Results are given as the average percentage (%) of cells expressing the respective marker and error bars represent the standard deviation. Shown is the result from one representative experiment performed in triplicate and 300 cells were evaluated for each marker and coverslip. (**D**) IB analysis of Npm1 in either undifferentiated (–diff) or differentiated (+diff) mouse NSCs. β-actin served as loading control. (**E**) IF staining of Npm1 (red) in undifferentiated (–diff) or differentiated (+diff) mouse NSCs. Right panel: merged (Npm1+DAPI) zoom-in of representative cells. (**F**) IF staining of NPM1 (red) in normal cortical rat astrocytes and in human glioma cell line U343MGa Cl2:6. (**G**) IB analysis of NPM1 levels in astrocytes and glioma cell lines U1242MG and U343MGa Cl2:6. β-actin served as loading control.

We first set out to determine the levels and localization patterns of NPM1 in astrocytic gliomas. We had previously validated the NPM1 monoclonal antibody FC82291. Immunoblotting (IB) and immunofluorescence (IF) staining using NPM1 depleted or siRNA control treated U2OS osteosarcoma cells, as well as wild type and *Npm1* knockout mouse embryo fibroblasts, showed that the antibody recognizes NPM1 specifically^31^. We applied this antibody for use in immunohistochemical (IHC) analysis of NPM1 in human glioma samples. A total of 60 cases of astrocytic gliomas were stained for NPM1 (16 grade I, 16 grade II, 15 grade III, and 13 grade IV) (Table 1). Nucleolar NPM1 immunoreactivity was detected in tumor cells of high and low grade gliomas in all 60 cases (Fig. 1A, Table 1). In low-grade tumors, some cells may appear negative, but a closer inspection revealed that NPM1 in these cells was mostly confined to nucleoli (Fig. 1B, Supplementary Fig. S1). NPM1 displayed increased staining in grade III and IV astrocytomas, whereas grade II astrocytomas presented NPM1 levels similar to grade I and adjacent near normal tissue (Fig. 1A,B, Table 1, Supplementary Fig. S1). NPM1 staining was most intense in grade IV tumors. Specifically, the staining intensity was significantly higher in Grade IV than in Grade I (*P* = 0.000245, chi square test), whereas no significant change in intensity was seen when comparing Grade I and Grade II (*P* = 0.674135), or Grade I and Grade III (*P* = 0.144893). However, a clear trend towards higher levels of NPM1 in grade III tumors when compared to grade I and II tumors was noted (Table 1, Fig. 1B). NPM1 staining in adjacent near normal areas of grade I and II tumors was similar to areas with more tumor cells (Supplementary Fig. S1), a finding in agreement with other studies^16^,^17^. We also scored NPM1´s subcellular localization pattern. Nucleolar NPM1 was detected in cells from all tumor samples regardless of grade. In grade I-III tumors the nucleoplasm of tumor cells was only modestly stained or not at all. In contrast, NPM1 was detected in the nucleoplasm besides the nucleolus in 12 out of 13 cases of grade IV tumors. We next investigated NPM1´s localization in a panel of human glioma cell lines. The U2OS osteosarcoma cell line served as an established control line for nucleolar NPM1^32^,^33^. We found that all glioma cell lines tested by us so far, including U343MGa Cl2:6, U343MG, U118MG, U178MG and U1240MG stained positive for NPM1 (Fig. 2A). NPM1 accumulated in nucleoli but a marked nucleoplasmic staining was also seen, similar to the grade IV tumors (Fig. 2A). A survey of The Cancer Genome Atlas data using the cBioPortal and the COSMIC databases revealed that no mutations in NPM1 have been found to date in 291 cases of high grade gliomas, according to exome and whole genome DNA sequencing. Hence in agreement with previous studies in glioma and other solid tumors, NPM1 is abundant and wild type^16^,^17^,^19^,^34^.

### Localization of Npm1 in neural stem cells and cortical astrocytes

Neural stem cells (NSCs) can differentiate into neurons, oligodendrocytes, and astrocytes when grown in medium containing serum or with certain defined growth factors. To gain further insights into the expression and localization of Npm1 we cultured mouse NSCs as neurospheres. The neurospheres were dissociated, split into 6-well plates and grown as single adherent cells. These cells were induced to differentiate or kept undifferentiated for 7 days. In the differentiation experiments, we obtained on average 17.3 ± 3.2% βIII-tubulin positive cells, 41.0 ± 16.1% GFAP positive cells, and 12.7 ± 4.0% CNPase positive cells (Fig. 2B,C). Nucleolar Npm1 was detected in both undifferentiated and differentiated NSCs (Fig. 2E). Npm1 was mainly localized to the nucleolus but we also noticed a more diffuse nucleoplasmic staining present in the undifferentiated cells, but not seen in the differentiated cells. IB analysis indicated a modest decrease in the levels of Npm1 in differentiated cells (Fig. 2D). Next, we compared the levels of NPM1 in glioma cells U1242MG and U343MGa Cl2:6 with normal cortical astrocytes from rat. A side-by-side comparison revealed markedly elevated levels of NPM1 in glioma cells when compared to astrocytes as shown by both IF staining and IB (Fig. 2F,G).

### Nucleolar re-organization in glioma cells with reduced NPM1

**Figure 3:**
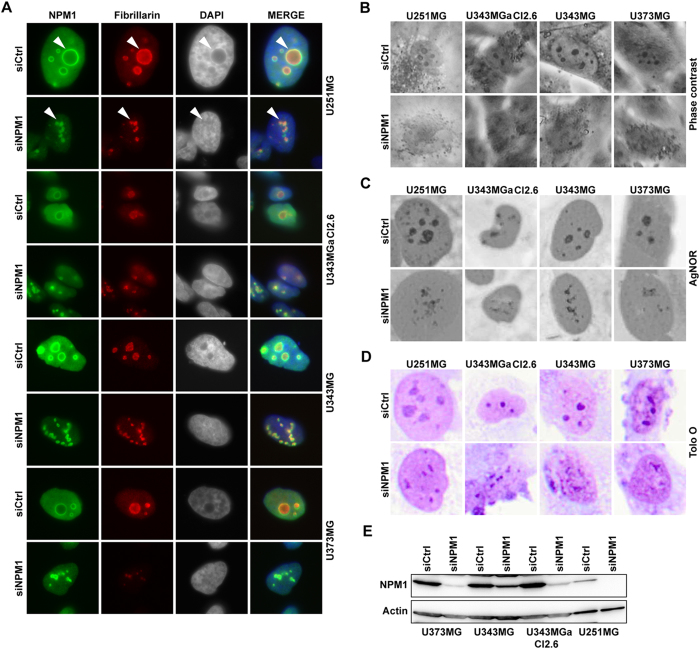
Knockdown of NPM1 in glioma cells alters nucleolar morphology. (**A**) IF staining of NPM1 (green) and fibrillarin (red) in glioma cell lines U343MGa Cl2:6, U373MG, U343MG, and U251MG treated with siNPM1 for 6 days. Nuclei were counterstained with DAPI. Arrowheads point at nucleoli. (**B**) Phase contrast micrographs of control and of cells depleted of NPM1. (**C**) AgNOR staining of control and NPM1 depleted glioma cells. (**D**) Acidic Toluidine Blue O staining of glioma cells. Nucleoli appear as darker stained than the surrounding nucleoplasm. (**E**) IB analysis of NPM1 levels in different glioma cell lines as indicated after siNPM1 treatment for 6 days. β-actin served as loading control.

**Figure 4:**
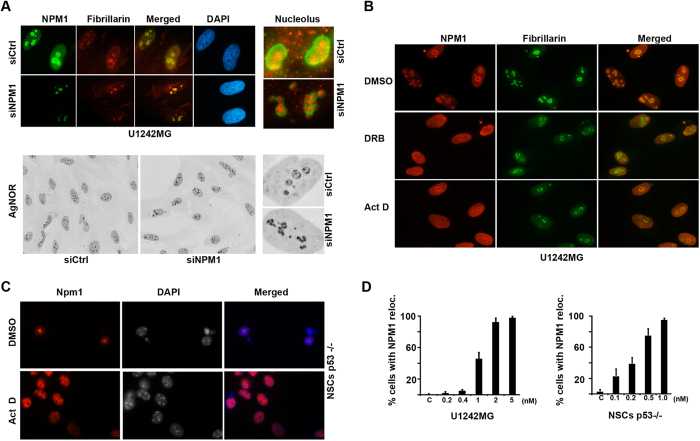
NPM1 re-distribution serves as a marker for nucleolar stress in glioma and neural stem cells. (**A**) Upper panel: U1242MG cells were transfected with either siCtrl or siNPM1 for 6 days and subsequently subjected to double IF staining for NPM1 (green) and fibrillarin (red). Nuclei were identified by DAPI. Lower panel: representative micrographs of AgNOR stained U1242MG cells either depleted of NPM1 or siCtrl treated. (**B**) U1242MG cells were treated with Act D (5 nM) or DRB (25μg/ml) for 8 hours and thereafter the cells were fixed, permeabilized, and IF stained for NPM1 (red) and fibrillarin (green). (**C**) IF staining of Npm1 in differentiated mouse p53^−/−^ NSCs. Nuclei were identified by DAPI. The NSCs were grown as spheres and thereafter dissociated onto laminin-coated coverslips and induced to differentiate. Npm1 (red) localizes to nucleoli in DMSO-treated cells but is increasingly localized to the nucleoplasm in Act D-treated cells. (**D**) Quantification of U1242MG cells (left panel) and mouse p53^−/−^ NSCs displaying NPM1 re-localization from the nucleolus to the nucleoplasm using different concentrations of Act D. Shown is the average percentage of cells from one representative experiment performed in triplicate. Error bars represent the standard deviation. In total 300 cells were evaluated per concentration and cell type.

We proceeded with silencing of NPM1 in the established human glioma cell lines using siRNA. Knockdown of NPM1 was detected on the 4^th^ day at the protein level. Therefore the assays were designed with observations usually on the 4^th^ day and beyond that. To first investigate the effects of NPM1 depletion on the nucleolus we performed double IF staining for NPM1 and the nucleolar protein fibrillarin in the cell lines U343MGa Cl2:6, U1242MG, U373MG, and U251MG (Fig. 3A). In NPM1 depleted cells, the nucleolar structure was transformed from symmetrical and round into a more fragmented and assymetrical morphology (Fig. 3A). Furthermore, phase contrast, silver (AgNOR), and acidic Toluidine Blue O staining each illustrated the changes in nucleolar morphology (Fig. 3B–D). Nucleoli appeared smaller in size and less phase dense while at the same time more numerous and asymmetrical. IB analysis confirmed a reduction in NPM1 in response to siNPM1 treatment (Fig. 3E). Identical changes in the morphology of the nucleolus were seen in the U1242MG cell line according to IF staining and AgNOR patterns (Fig. 4A). Specifically, U1242MG cells presented with 72.4 ± 6.5% round and compact nucleoli in cultures transfected with siCtrl but only 7.1 ± 2.7% round nucleoli in siNPM1-treated cells (*P* < 0.001, two-tailed t-test). Nucleoli stain poorly with the fluorescent dye DAPI whereas perinucleolar heterochromatin is intensely stained^35^. Depletion of NPM1 in glioma cell lines resulted in less visible nucleoli and altered chromatin structure around nucleoli as seen by DAPI (Supplementary Fig. S2). To investigate if NPM1 silencing had any effect on glioma cell proliferation we selected three glioma cell lines that were subjected to transfection with NPM1 siRNA. Cell cycle analysis by FACS using propidium iodide labeling of DNA, indicated no major change in cell cycle phase distribution in U1242MG and U251MG glioma cells when depleted of NPM1 (Supplementary Fig. S3). A modest shift was seen in U343MGa Cl2:6 cells with more cells in S and G2 phase and fewer cells in G1 in the cultures treated with siNPM1. Only minor alterations in the cell proliferation rate were seen when comparing control and NPM1 depleted cells as assessed by the MTT assay (Supplementary Fig. S3). Although NPM1 was not completely silenced we conclude that the bulk of NPM1 protein is dispensable for glioma cell proliferation in the shorter term.

### NPM1 serves as a marker for nucleolar stress in glioma cells and NSCs

The nucleolus can reorganize itself in many circumstances, including mitotic disassembly and reassembly, or when exposed to chemicals such as actinomycin D (Act D) or the adenosine analogue 5,6-dichloro-β-D-ribofuranosyl-benzimidazole (DRB)^36^,^37^. In particular Act D has been used as a tool to induce nucleolar disruption due to inhibition of RNA pol I^38^. DRB is on the other hand an inhibitor of RNA pol II transcription causing segregation of the nucleolar compartments^38^. To investigate any resemblance between the structural changes during NPM1 depletion and inhibition of DNA transcription, we analyzed the localization of fibrillarin, a marker of the nucleolar dense fibrillar centers in cells treated with Act D (5nM) or DRB (25 μM). Following inhibition of RNA pol II with DRB, the nucleoli of U1242MG cells appeared elongated and fibrillarin rich areas were disorganized and more diffuse compared to control cells (Fig. 4B). When RNA polymerase I activity was inhibited by Act D, nucleoli adopted a spherical smaller structure and with fibrillarin rich aggregates at their periphery, also known as nucleolar caps^39^ (Fig. 4B). We noted that nucleoli in cells depleted of NPM1 did not at all resemble nucleoli in cells exposed to Act D. Some resemblance with DRB was noted, for example a subset of the NPM1 depleted cells and some of the DRB treated cells presented with a so called “beaded necklace structure”. A complete segregation of nucleolar sub-compartments as seen in the case of DRB treated cells was not found in NPM1 depleted cells.

Re-localization of NPM1 from the nucleolus to the nucleoplasm is a hallmark of nucleolar stress following inhibition of RNA Pol I^40^. Less is however known about the dynamics of NPM1 in glioma cells and neural stem cells when they are exposed to a low concentration of Act D. To investigate the effects of RNA pol I inhibition on NPM1 localization, we treated differentiated mouse NSCs and a human glioma cell line U1242MG with Act D. To obtain comparable results, since U1242MG harbors mutant p53 we used *Trp53*^*−/−*^ NSCs in these experiments. NPM1 staining intensity in the nucleoplasm increased in U1242MG cells treated with Act D, while the nucleolar staining diminished (Fig. 4B). Majority of the cells displayed NPM1 re-localization and an altered nucleolar shape at concentrations above 2 nM (Fig. 4D). Upon treatment with Act D, the marked nucleolar accumulation of Npm1 in *Trp53*^*−/−*^ NSCs was disrupted and instead replaced by a more homogenous nuclear Npm1 staining (Fig. 4C). The NSCs responded to Act D at a much lower concentration and more than 50% of the NSCs displayed Npm1 re-localization at 0.5 nM (Fig. 4D). Concentrations of 2 nM and above rapidly became cytotoxic to the NSCs, and analysis of cell morphology (nuclear structure) was therefore not feasible. We conclude from these experiments that NPM1 is a suitable marker for nucleolar stress in NSCs and glioma cells.

### NPM1 influences survival of glioma cells treated with Actinomycin D

**Figure 5:**
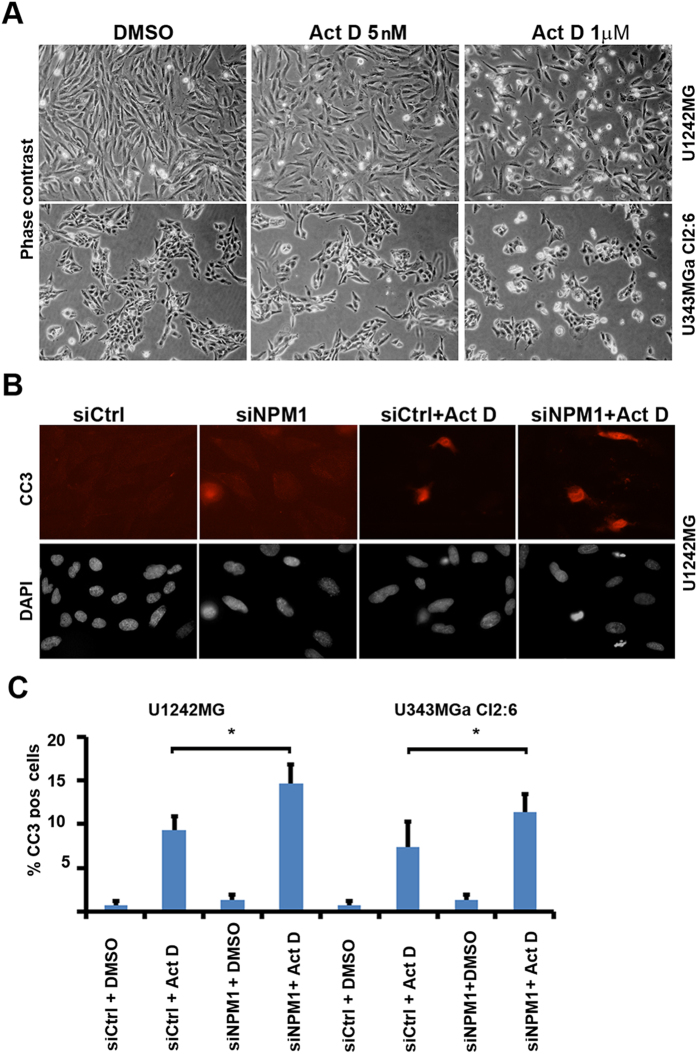
NPM1 influences sensitivity to actinomycin D induced glioma cell apoptosis. (**A**) Phase contrast photomicrographs of DMSO (control) and Act D treated U1242MG and U343MGa Cl2:6 cells. Cells were treated for 24 hours. (**B**) IF staining for cleaved caspase 3 (CC3, red). Nuclei were counterstained with DAPI (grey). Apoptosis is increased in U1242MG cells silenced for NPM1 and then challenged with Act D (5 nM) for 24 hours. The cells were transfected with either siNPM1 or siCtrl for 4 days prior to the addition of Act D or DMSO. (**C**) Quantification of (%) CC3^+^ U1242MG and U343MGa Cl2:6 cells. Shown is the average percentage of CC3^+^ cells for each treatment category stemming from three independent biological replicates in triplicate. Error bars represent standard deviation and statistical significance is indicated (*P < 0.05, two-tailed t-test). Approximately 1000 cells were evaluated for each condition.

We hypothesized that reduced levels of NPM1 may sensitize glioma cells to cell death, while the abundant NPM1 would protect the glioma cells from death signals (apoptosis). We treated growing cultures of U1242MG and U251MG cells with various concentrations of Act D as indicated. We did not observe any increase in cell death in either line when exposing the cells to 5 nM Act D for 24 hours (Fig. 5A). A higher concentration of Act D (1 μM), known to inhibit both RNA pol I and RNA pol II^41^,^42^, triggered cell death as observed by phase contrast microscopy (Fig. 5A). Depletion of NPM1 increased the number of cleaved caspase-3+ cells following exposure to Act D for 24 hours, which was evident in both U251MG and U1242MG cell cultures, whereas depletion of NPM1 only did not trigger an increase in apoptosis (Fig, 5B,C). We also wanted to determine the effect of NPM1 silencing on the glioma cells response to the more clinically relevant compounds temozolomide (TMZ) and 5-fluorouracil (5-FU). An overall reduction in NPM1 did not enhance U343MGa Cl2:6 or U1242MG cell death by TMZ or 5-FU treatment as assessed by a fluorometric cytotoxicity assay (Supplementary Fig. S3). It should be noted that the knockdown of NPM1 in U343MGa Cl:2:6 was only partial, and the compounds TMZ and 5-FU had to be used at very high concentrations in order to have some cytotoxic effects on these lines. For example, U343MGa Cl2:6 was barely affected by TMZ and this may mask any potential pro-survival function of NPM1 (Supplementary Fig. S3).

### Silencing of linker histone H1.5 triggers apoptosis in glioma cells that is modulated by NPM1

**Figure 6:**
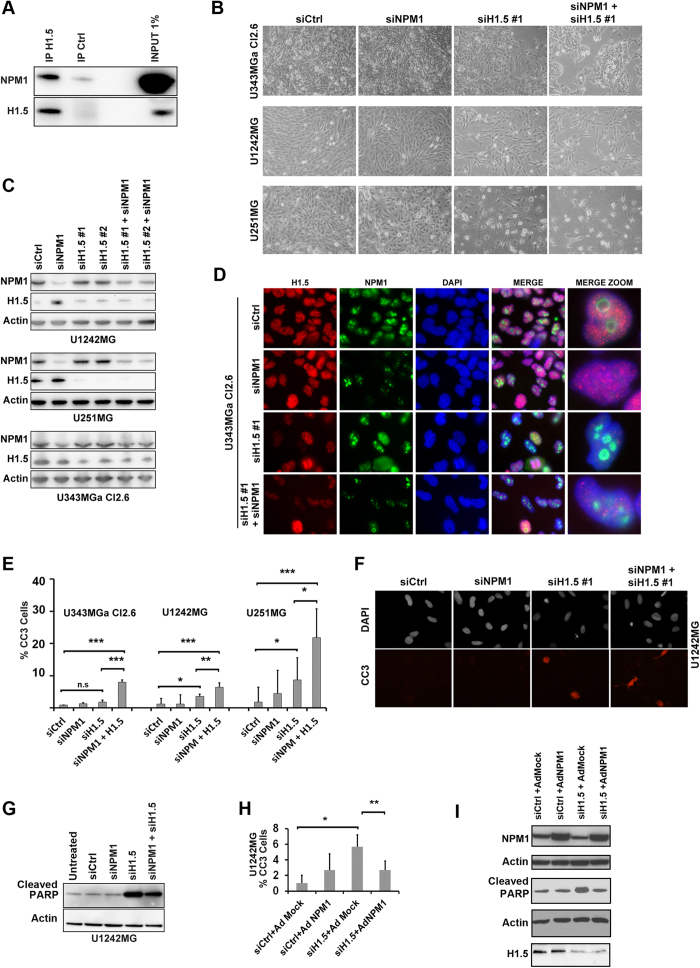
Depletion of linker histone H1.5 induces apoptosis in glioma cells and this is modulated by NPM1. (**A**) Identification of linker histone H1.5 as an NPM1 associated protein. H1.5 co-immunoprecipitation showed increased levels of NPM1 bound to H1.5 when compared to isotype (IgG antibody) control according to IB analysis. (**B**) Morphological appearance of glioma cell cultures (U343MGa Cl2:6, U1242MG, and U251MG) depleted of NPM1, H1.5 or a combination. Cell cultures were transfected with siRNA targeting NPM1 (siNPM1 pool) or two different siRNA pools targeting H1.5 (siH1.5 #1 and siH1.5 #2), or combinations as indicated. For experiments in panels B-G the following time line was used: cells were treated with siNPM1 for an initial 3 days after which cells were further treated with siNPM1 and siH1.5 alongside controls for 3 days. (**C**) Knockdown of NPM1 and H1.5 in glioma cells as evidenced by IB analyses. (**D**) Knockdown of NPM1 and H1.5 in glioma cell line U343MGa Cl2:6 visualized by IF staining for H1.5 (red), NPM1 (green), and DAPI (blue). Far right panel shows zoom-in of cells representing specific cell phenotypes. (**E**) Quantification of (%) cells expressing cleaved caspase 3 (CC3) in control, siNPM1, siH1.5 and siNPM1+siH1.5 treated glioma cell lines. (**F**) IF staining of cleaved caspase 3 (CC3, red) and DAPI in cells treated with siNPM1 and siH1.5. (**G**) IB analysis of the amounts of cleaved PARP in cells treated with siNPM1, siH1.5 or a combination. β-actin served as loading control. (**H**) U1242MG cell cultures infected with NPM1 (Ad-NPM1) show decreased caspase-3 activation when compared with mock (Ad-Mock) infected cells. Shown is the average percentage of CC3^+^ cells for each treatment category from three independent biological replicates each in triplicate. Error bars represent standard deviation and statistical significance is indicated. Statistical analysis was done according to one way analysis of variance (ANOVA) with Tukey-Kramer post-hoc test (*P < 0.05, **P < 0.01, ***P < 0.001). (**I**) IB analyses of NPM1, cleaved PARP, and H1.5 levels in U1242MG cells, corresponding to the experimental set-up used in the H panel. β-actin served as loading control, note that two separate blot membranes were used.

Using nuclear complex co-immunoprecipitation (co-IP) we had previously identified a number of NPM1 binding proteins in the glioma cell line U1242MG^31^. One of the proteins identified was linker histone H1.5. Histone H1 interacts with linker DNA between nucleosomes and functions in the compaction of chromatin into higher order structures^43^. Humans and mice have eight histone H1 genes and among them all of the somatic H1s (H1.1–H1.5) are ubiquitously expressed in all body tissues throughout development^43^,^44^. Six peptides matching H1.5 were identified by mass spectrometry and a database search revealed that the sequences of two of these peptides perfectly matched H1.5, but none of the other forms^31^ (Supplementary Fig. S4). Nuclear complex co-IP of H1.5 (IP with H1.5 antibody) showed increased amounts of NPM1 compared to isotype control co-IP confirming the association between NPM1 and H1.5 (Fig. 6A). Loss of H1.5 has been shown to induce cell cycle arrest and apoptosis^44^,^45^. Given that both NPM1 and H1.5 have been implicated in apoptosis we decided to further study any potential interplay between these two proteins in glioma cells. U343MGa Cl2:6, U1242MG and U251MG cells were treated with siNPM1 for an initial 3 days after which the cells were further treated with siNPM1 and siH1.5 alongside controls. Visual inspection revealed that there were no major changes in the overall morphological appearance of the U1242MG, U251MG, and U343MGa Cl2.6 cell cultures when depleted of NPM1 (Fig. 6B). A clear reduction in cell density was seen in cultures depleted of H1.5 and in cultures co-depleted of both NPM1 and H1.5 (Fig. 6B). Using an antibody specific for the H1.5 isoform we confirmed that the protein is present in glioma cells and predominantly localized throughout the nucleus. Depletion of NPM1 and H1.5 protein using siRNA was confirmed by IB analysis (Fig. 6C) as well as IF staining (Fig. 6D). H1.5 expression and knockdown was verified by qRT-PCR (Supplementary Fig. S5), and depletion was more efficient in U251MG than in U1242MG.

Nuclear counterstaining with DAPI indicated DNA condensation in a subset of cells depleted of H1.5 indicative of apoptosis. To further investigate this, glioma cell lines U343MGa Cl2:6, U1242MG and U251MG were analyzed for cleaved caspase 3 (Fig. 6E,F). Quantification of cells expressing cleaved caspase 3 in control, siNPM1, siH1.5 and siNPM1+siH1.5 treated glioma cell lines showed a significant increase in the number of U1242MG (*P* < 0.05) and U251MG cells (*P* < 0.05) that stained positive for cleaved caspase 3 in cells treated with siH1.5 in comparison to siCtrl, although there was not significant difference in U343MGa Cl2:6 (Fig. 6E). Depletion of NPM1 alone did not increase apoptosis. There was an increase in the percentage of cells that stained positive for cleaved caspase 3 in cells treated with siNPM1 in combination with siH1.5 in comparison to cells treated with siH1.5 alone, and this was significant in all three cell lines (Fig. 6E,F). Thus, depletion of H1.5 triggered apoptosis and when combined with a reduction in NPM1, the apoptosis was more pronounced.

To confirm apoptosis we also evaluated cellular amounts of cleaved PARP by IB. Depletion of H1.5 increased the amount of cleaved PARP although any further increase in the level of cleaved PARP was not seen when cells were co-depleted of H1.5 and NPM1, although minor differences can´t be excluded (Fig. 6G). The reason for this discrepancy between PARP and cleaved caspase-3 is unclear but PARP1 is a nucleolar protein and also a major NPM1 binding partner^46^. To investigate this further, we counted the number of rounded up cells with signs of membrane blebbing as seen under the phase contrast microscope. We counted 5.3 ± 2.6% cells with membrane blebbing in cultures of U1242MG treated with siH1.5#1, whereas in cultures treated with siH1.5#1+siNPM1 we counted 9.8 ± 2.1% cells (*P* < 0.05, two-tailed t-test).Thus the fraction of rounded up cells with membrane blebbing better matched the CC3^+^ cell numbers than amounts of cleaved PARP. We next addressed a potential anti-apoptotic effect of NPM1 by infecting H1.5 depleted U1242MG cell cultures with adenovirus (Ad-NPM1). Upon enforced virus mediated expression of NPM1 in H1.5 depleted cells there was a significant reduction in the percentage of CC3^+^ cells (from 5.67 ± 1.53% down to 2.67 ± 1.15%; *P* < 0.01) (Fig. 6H). Levels of NPM1, knockdown of H1.5, and amount of PARP cleavage was assessed by IB (Fig. 6I). The protective effect was not observed in H1.5 depleted cell cultures when using an empty adenovirus (Ad-Mock), while introduction of NPM1 restored the enhanced PARP level in H1.5 depleted cells back to normal. We conclude that silencing of H1.5, but not of NPM1, triggers apoptosis in glioma cell lines. However, NPM1 modulated the apoptotic response in H1.5 depleted cell cultures.

## Discussion

The role of NPM1 in cancer development remains complex and NPM1 has been ascribed both pro-tumorigenic and tumor suppressive functions^9^. NPM1 has to date not been directly shown to affect glioma initiation and progression, although implicated in cellular signaling downstream of mTOR with an impact on the dynamics of the cytoskeleton of astrocytes^47^. Herein, we confirmed recent reports showing that NPM1 is highly expressed in glioblastomas^16^,^17^,^18^,^19^. NPM1^+^ cells were detected in low-grade astrocytic gliomas and in adjacent near-normal brain areas, but the staining intensity was considerably weaker than in glioblastomas. This is in line with a study showing increased expression of NPM1 in grade III and IV tumors, while grade II astrocytomas were found to be similar to non-neoplastic brain tissue^17^. High NPM1 is associated with short survival in glioblastoma but it does not seem to have a high prognostic value^18^. We also evaluated NPM1´s subcellular localization patterns. NPM1 was concentrated to glioma cell nucleoli, but a marked nucleoplasmic NPM1 staining was observed in the majority of grade IV tumors, in human glioma cell lines, as well as in undifferentiated mouse NSCs. In contrast, predominantly nucleolar staining was seen in the human low-grade tumor samples, differentiated mouse NSCs and in rat cortical astrocytes. Hence, we emphasize here that NPM1 is not an exclusive nucleolar marker protein. Besides elevated levels of the protein resulting in a nucleolar spillover, it is possible that the chromatin landscape in glioblastoma cells and NSCs may allow for an increase in nucleoplasmic NPM1. A third possibility is that glioblastoma cells and NSCs are exposed to more cellular damage, including nucleolar stress, creating a shift in NPM1´s localization more towards the nucleoplasm.

We found that NPM1 depletion triggered a distortion of the nucleolar structure in glioma cells suggesting that NPM1 is critical to maintain the round, fairly symmetrical, nucleolar structure in glioma cells in agreement with studies performed using other cell types^31^,^48^,^49^. Disruption of chromatin organization and nucleolar morphology may in turn contribute to genome instability^50^. Decreased cell proliferation rates and increased apoptosis in response to NPM1 depletion have been reported^27^,^32^,^51^,^52^,^53^. However it was noted that U87MG glioma cell proliferation rate did not change significantly in response to NPM1 knockdown until after the 7^th^ day of transfection^54^. In agreement, we found that a reduction in NPM1 levels did not have any major effects on glioma cell viability or cell proliferation despite alterations in nucleolar morphology. NPM1 depleted glioma cell cultures remained viable. It is important to keep in mind that NPM1 is a stable protein with a half-life of up to ~30–40 hours^55^, and the interpretation of our experimental findings should take into consideration the presence of some residual NPM1 protein in the cells. Notably, mouse embryo fibroblasts deficient in both *p53* and *Npm1* are viable and proliferate *in vitro*, indicating that Npm1 is not essential to sustain cell proliferation^22^.

The ability of NPM1 to suppress apoptosis may play a significant pro-survival role during tumor development^9^. It was reported that loss of NPM1 sensitized U87MG and A172 glioma cells to temozolomide (TMZ) with an increase in cell death^54^. The glioma cell lines we used appeared relatively resistant to TMZ. We therefore induced nucleolar stress and apoptosis by treating the cells with a high concentration (1 μM) of Act D. Cytotoxicity triggered by a high concentration of Act D is mediated by multiple mechanisms including inhibition of *both* RNA pol I and II transcription causing nucleolar stress that consequently leads to the release of a number of proteins into the nucleoplasm including NPM1^56^. This re-localization of NPM1 to the nucleoplasm of glioma cells and NSCs induced by Act D was easily detected here. We found that glioma cell apoptosis induced by Act D was significantly increased in the context of reduced levels of NPM1. In this setting, NPM1 may have pro-survival effects on a molecular level related to maintenance of the nucleolar structure, but such mechanisms may also operate in a nucleolus-independent manner. Further studies are needed to elucidate this. Unfortunately, inefficient viral shRNA mediated knockdown of Npm1 prevented us from specifically assessing its role in differentiation and in the nucleolar stress response of mouse NSCs.

We explored the pro-survival role of NPM1 further. We had previously identified the linker histone H1.5 isoform as an NPM1 interacting protein in glioma cells^31^. NPM1 is also a proposed chaperone for H1 linker histone^57^. Linker histones have been implicated in apoptosis. For example, DNA damage mediates the release of histone H1.2, inducing cytochrome c release and leading to apoptosis^58^. The cytochrome c-releasing ability of H1.2 was shown to be unique, since the other four components (H1.1, H1.3-H1.5) of histone H1 did not show strong cytochrome c-releasing activity^58^. Interestingly, genes reported to be upregulated in H1.5 depleted cells were enriched for involvement in cell death and apoptosis^45^. From our experiments we could conclude that silencing of H1.5, but not NPM1, triggered apoptosis in glioma cell lines. A reduction in NPM1 modestly sensitized glioma cells to cell death triggered by a deficiency in H1.5 suggesting that NPM1 may act in a pro-survival manner. While our results demonstrate a link between NPM1 and linker histone H1.5 in the regulation of cell death it remains to be determined how this occurs. Since NPM1 depletion also sensitizes glioma cells to Act D it might be suggested that this is due to accumulated targeting of DNA integrity rather than a specific loss of function as a consequence of a lost NPM1-H1.5 interaction, this remains to be determined. In summary, NPM1 was significantly upregulated in glioblastomas and displayed a more generalized nuclear staining compared with the more exclusive nucleolar staining seen in normal cells. While we did not observe any major effects on glioma cell proliferation and viability by NPM1 depletion only, our results suggest that NPM1 act in a pro-survival manner when cells are stressed.

## Methods

### Cell culture

Glioma cell lines U343MG, U373MG, U251MG, U343MGa Cl2:6, U118MG, U178MG, U1240MG, U1242MG, and U87MG have been previously characterized^59^,^60^. Two cell lines (U343MG and U343MGa Cl2:6) with different phenotypic characteristics were initially established from the same glioblastoma biopsy^61^. Cells were cultured in DMEM containing 10% fetal bovine serum, antibiotics (100 μg of penicillin and 50 μg of streptomycin sulfate/mL), and 2 mM glutamine at 37 °C, 5% CO_2_.

### Animals

The animal experiments were approved under ethical permit N90/11 (Stockholms Norra Djurförsöksetiska nämnd avd. 1) and in accordance with Karolinska Institutet´s approved guidelines for animal experimentation. The wild type (wt) mouse strain used was C57Bl6/J (Jackson laboratory) and the p53 null mouse strain was *Trp53*^*−/−*^ (TSG-p53) mice (Taconic, NY, USA). Mouse embryos were collected at the stage E14.5 with the assumption that mating occurred at midnight with plug detection before the following noon. Adult mice were 2-3 months old.

### Neural stem cell and astrocyte culture

Ganglionic eminence and subventricular zone were isolated from embryonic wt mice, adult wt and p53 null mice, respectively. Tissues were mechanically dissociated using a P-1000 pipette. Cells were then plated at a density roughly of 500000-1000000 cells in Corning^®^ Ultra-Low attachment cell culture flasks 25 or 75 cm^2^ in Stemcell Technologies NeuroCult^®^ NS-A Proliferation Kit (Mouse) supplemented with 2 μg/ml Heparin, EGF 20 ng/ml and bFGF 20 ng/ml. Cells were continuously given new media and growth factors weekly and passaged every one to two weeks dependent on growth rate. Cell differentiation was performed by plating cells in Stemcell Technologies NeuroCult^®^ NS-A Differentiation Kit (Mouse) on Laminin-1 (Sigma) coated (100 μg/ml Laminin-1/PBS for 1 hour at 37 °C) glass slides (22×22 mm) in 6-well plates. Rat cortical astrocytes (Life technologies) were grown as adherent monolayer culture in DMEM with 10% FBS and thereafter fixed and processed for IF.

### Drug treatments

Compounds were purchased from Sigma Aldrich. Actinomycin D was dissolved in DMSO as a stock solution of 1 mg/ml. The adenosine analogue 5,6-dichloro-1-β-D-ribofuranosylbenzimidazole (DRB) was dissolved in ethanol (95%) and used at a final concentration of 25 μg/ml.

### Transfection of siRNA oligonucleotides

Cells growing in 6-well plastic plates were transfected with oligofectamine according to the manufacturer´s instructions (Life Technologies). siRNAs were purchased from Dharmacon including: siGENOME On-TARGETplus SMARTpool human NPM1 (#4869, denoted as siNPM1); siGENOME Human HIST1H1B/H1.5 (#3009) siRNA SMARTpool (denoted as siH1.5#1); and siGENOME non-targeting siRNA pool #1 (D-001206-13-05) denoted as siCtrl. HIST1H1B/H1.5 Stealth siRNA HSS142366 (siH1.5#2) was from ThermoFisherScientific. To reduce NPM1 protein level it was necessary to conduct two rounds of siRNA transfections four days apart.

### Viral infection

Cells were transfected with H1.5 siRNA 24 hours prior to the start of infection. Cells were infected with adenovirus in DMEM media supplemented with 2% FBS and were incubated for two hours in a 37 °C incubator with 5% CO_2_. Infected cells were then washed with pre-warmed PBS and replaced with fresh DMEM supplemented with 10% FBS until cell lysis or fixation for immunostainings. Pre-made null control and NPM1 adenovirus ~1×10^11^ infectious units /ml in PBS were from SignaGen Laboratories (MD, USA).

### Measurements of cell death

Visual inspection of cell cultures and nuclear counterstaining with DAPI was used to observe membrane blebbing and DNA condensation respectively. Cleaved caspase-3 (CC3) positivity was determined by IF staining. The CC3 antibody detects endogenous levels of the large fragment (17/19 kDa) of activated caspase-3 resulting from cleavage adjacent to Asp175. The antibody does not recognize full length caspase-3. Cleaved PARP was detected by IB and IF staining. The rabbit monoclonal [E51] to cleaved PARP is specific for the 25 kDa cleaved form of human PARP.

### Immunoblotting and immunoprecipitation

Detailed procedures for analysis of total and detergent soluble proteins, co-IP, and IB have been published^31^.

### Immunofluorescence staining and microscopy

IF staining of cells and microscopy was conducted as described^31^.

### Antibodies

Primary antibodies used were: NPM1 (mouse IgG1, clone FC82291, Sigma-Aldrich); β-actin (mouse IgG1, clone AC-15, Sigma-Aldrich); fibrillarin (rabbit, ab 5821 Abcam); cleaved caspase-3 at Asp175 (mouse, Cell Signaling); anti-cleaved PARP (rabbit MAb, clone E51, ab 32064, Abcam); H1.5 (rabbit, ab24175, Abcam); Glial fibrillary acidic protein (GFAP, Sigma-Aldrich); β-Tubulin III (TUJ1 clone, Covance); CNPase (monoclonal IgG1, clone 11-5B, Sigma-Aldrich). Secondary antibodies were: anti-rabbit and anti-mouse HRP conjugated antibodies (Amersham); anti-mouse and anti-rabbit conjugated with FITC or Texas Red (Vector).

### AgNOR staining

Silver staining of the nucleolus organizer regions (AgNOR staining) was conducted as described^38^. Toluidine Blue O staining of glioma cell lines was also conducted as described^31^,^38^.

### Statistics and bioinformatics

Experiments were conducted in three independent experiments (biological replicates), each in triplicate, and results presented as the mean ± standard deviation (SD) unless stated differently in the figure legends. Student’s *t*-test (two-tailed) was used to evaluate the differences in the data between two groups. Statistical analysis with 3 or more groups was performed with one-way analysis of variance (ANOVA) and Tukey-Kramer post-hoc test for each pair of means. For analysis of the NPM1 IHC scores in Table 1 that were treated as categorical data, we used the non-parametric Chi-Square test. Mean, SD and *t*-test were calculated in Microsoft Excel. Statistical analysis with the Chi Square test and ANOVA was performed using Graphpad Prism v 5.0 software. Statistical probability (*P*) was set at *P < 0.05, **P < 0.01 and ***P < 0.001. The cBioPortal and COSMIC databases was used to survey TCGA data for mutations in *NPM1*. Sequence alignments were made in Clustal W.

### Tissue specimens and immunohistochemistry

A total of 60 cases of astrocytic gliomas, including 16 grade I, 16 grade II, 15 grade III and 13 grade IV tumors were collected in Stockholm, at Karolinska University Hospital in Solna between the years 2004 and 2007. The specimens were formalin-fixed and paraffin embedded, anonymized for use in this study. The use of the specimens had been approved by the regional ethical committee in Stockholm (permit no. 2005/542-31/1). Collaborating pathologist Abiel Orrego reviewed all haemotoxylin and eosin-stained slides of the samples to confirm diagnosis and grading. Immunostainings were performed using the Ventana Discovery Automated Staining machine following the vendor´s suggestions (Ventana Medical Systems, Tucson, Arizona). Removal of paraffin was done in the Ventana machine and the heat-induced epitope retrieval method in Tris-Borate EDTA buffer pH8.0 was used. A streptavidin-biotin horseradish peroxidase based DAB kit provided by Ventana was used for detection in the case of NPM1 staining. Sections were counterstained with hematoxylin. Finally the slides were rehydrated in graded ethanol rinses, cleared in xylene and mounted in pertex. Each section was examined under x100 to x400 magnifications using an Olympus light microscope (UPMTVC Japan) and a coupled Leica camera (DFC320) was used for documentation. First, the NPM1 immunostained astrocytoma samples were classified as negative (<10% NPM1 stained cells) or positive (>10%), and all samples were positive. We therefore scored NPM1 according to intensity of staining as follows: weakly positive (+), moderately positive (++), strongly positive (+++), and those with intense/saturated staining (++++). Nucleolar, nucleoplasmic and cytoplasmic NPM1 staining was scored independently.

## Additional Information

**How to cite this article**: Holmberg Olausson, K. *et al.* NPM1 histone chaperone is upregulated in glioblastoma to promote cell survival and maintain nucleolar shape. *Sci. Rep.* **5**, 16495; doi: 10.1038/srep16495 (2015).

## Supplementary Material

Supplementary Information

## Figures and Tables

**Table 1:** NPM1 immunoreactivity in human astrocytic gliomas.

	Grade I, n (%)	Grade II, n (%)	Grade III, n (%)	Grade IV, n (%)
+	5 (33.3)	6 (37.5)	1 (6.0)	0 (0.0)
++	4 (26.6)	5 (31.5)	4 (18.7)	0 (0.0)
+++	6 (33.3)	3 (18.75)	5 (37.5)	2 (15.0)
++++	1 (6.6)	2 (12.5)	5 (37.5)	11 (85.0)

NPM1 immunostained astrocytoma samples were initially classified as negative (<10% NPM1 stained cells) or positive (>10%), and all samples were found to be NPM1 positive. The NPM1 IHC was then scored according to intensity of staining as follows: weakly positive (+), moderately positive (++), strongly positive (+++), and those with intense/saturated staining (++++). n = number of cases, expressed as percentage in parenthesis.
