# Supplementary material for: NPM1 histone chaperone is upregulated in glioblastoma to promote cell survival and maintain nucleolar shape
Source: Sci Rep. 2015 Nov 12;5:16495. doi: 10.1038/srep16495 (PMC4642306; doi:10.1038/srep16495)
Supplement: Supplementary Information [file srep16495-s1.pdf]

SUPPLEMENTAL FILES

NPM1 histone chaperone is upregulated in glioblastoma to promote cell survival and maintain nucleolar shape

Karl Holmberg Olausson[1,*], Tamador Elsir[1,2], Kaveh Moazemi Goudarzi[1], Monica Nistér[1] and Mikael S. Lindström[1,3 *]

[1]Department of Oncology-Pathology, Karolinska Institutet, Cancer Center Karolinska, CCK R8:05, Karolinska University Hospital in Solna, SE-17176 Stockholm, Sweden
[2]Department of Neuroscience, Neurology, Uppsala University, Uppsala University Hospital, SE-75185 Uppsala [3]Science for Life Laboratory, Division of Translational Medicine and Chemical Biology, Department of Medical Biochemistry and Biophysics, Karolinska Institutet, Stockholm, Sweden, Mikael.Lindstrom@ki.se

Supplemental methods

DNA content analysis by FACS

Monolayer cells were trypsinized and washed 1x with PBS in a 15ml Conical tube, spun down at 1,500rpm for 5 min and re-suspended in 1ml 1x PBS. Fixation of cells was done by adding two volumes (2ml) of cold (-20°C), absolute (100%) ethanol, while vortexing. Thereafter the conical tubes were filled with 1x PBS, and after mixing the cells were precipitated by centrifugation at 1,500rpm for 5 min. Cells were washed once with 1x PBS and again spun down at 1,500rpm for 5 min followed by re-suspension in 0.5-1ml (depending on the number of cells) staining solution [1x PBS containing 50µg/ml PI, 0.1mg/ml RNase A], and incubated for at least 30 minutes at 4°C in the dark. Cell cycle profiles were obtained on a FACScan™II flow cytometer (Becton Dickinson BD, Franklin Lakes, NJ) and analyzed using the BD CellQuestPro™ software.

MTT assay

The MTT (3-[4,5-dimethylthiazol-2-yl]-2,5 diphenyl tetrazolium bromide) assay was used to measure the relative fraction of viable cells and this was done according to the manufacturer´s instructions (Sigma-Aldrich). For the MTT assay we used cells that had been either depleted of NPM1 or treated with siCtrl for 4 days prior to the start of the assay. Equal numbers of cells were seeded in triplicates with separate plates for each time point and cell line. Plates were incubated with MTT for 4 hours and the absorbance measured by spectrophotometer at 590 nm.

Fluorimetric cytotoxicity assay

Cells plated at a 25% confluence were treated with siRNA the following day as described above. On day 3 the cells were split and seeded onto 96-well microtiter plates, incubated overnight (reaching a 25% confluence) and then re-transfected with siRNA. On day 5 of siRNA treatment media was removed and substituted with media containing either 1mM Temozolomide, 1mM 5-Fluorouracil or DMSO as control (compounds were from Sigma Aldrich). Each condition was run as triplicate. Following

siRNA and drug treatment fluorimetric cytotoxicity assay was performed. After 24 hours incubation, the medium was removed and the cells were washed in PBS. Fluorescein diacetate (FDA, Sigma) was dissolved in DMSO and kept frozen at -20°C as a stock solution (10 mg/ml). The FDA stock solution was diluted in PBS at a concentration of 10µg/ml and 200µl was added to each well. The plates were then incubated for 30 min at 37°C. A 96-well scanning fluorometer (Tecan Infinite M1000) was used to count the emitted fluorescence.

Quantitative real time PCR

Total cellular RNA was extracted with TRIzol® reagent (Life Technologies). Real-time quantitative reverse transcriptase-polymerase chain reaction (qRT-PCR) was performed to monitor H.1.5 mRNA expression. The Power SYBR® Green RNA-to-C_T™ 1-Step kit was used in conjunction with an Applied Biosystems 7500 Real-Time PCR system. GAPDH was used as the internal standard reference. We used primer pairs unique to H1.5 F1´ AAATCCCCGGCTAAGAAGAA R1´ and F2´ TCTTAAGGGCTGCCAAAGAA R2´.

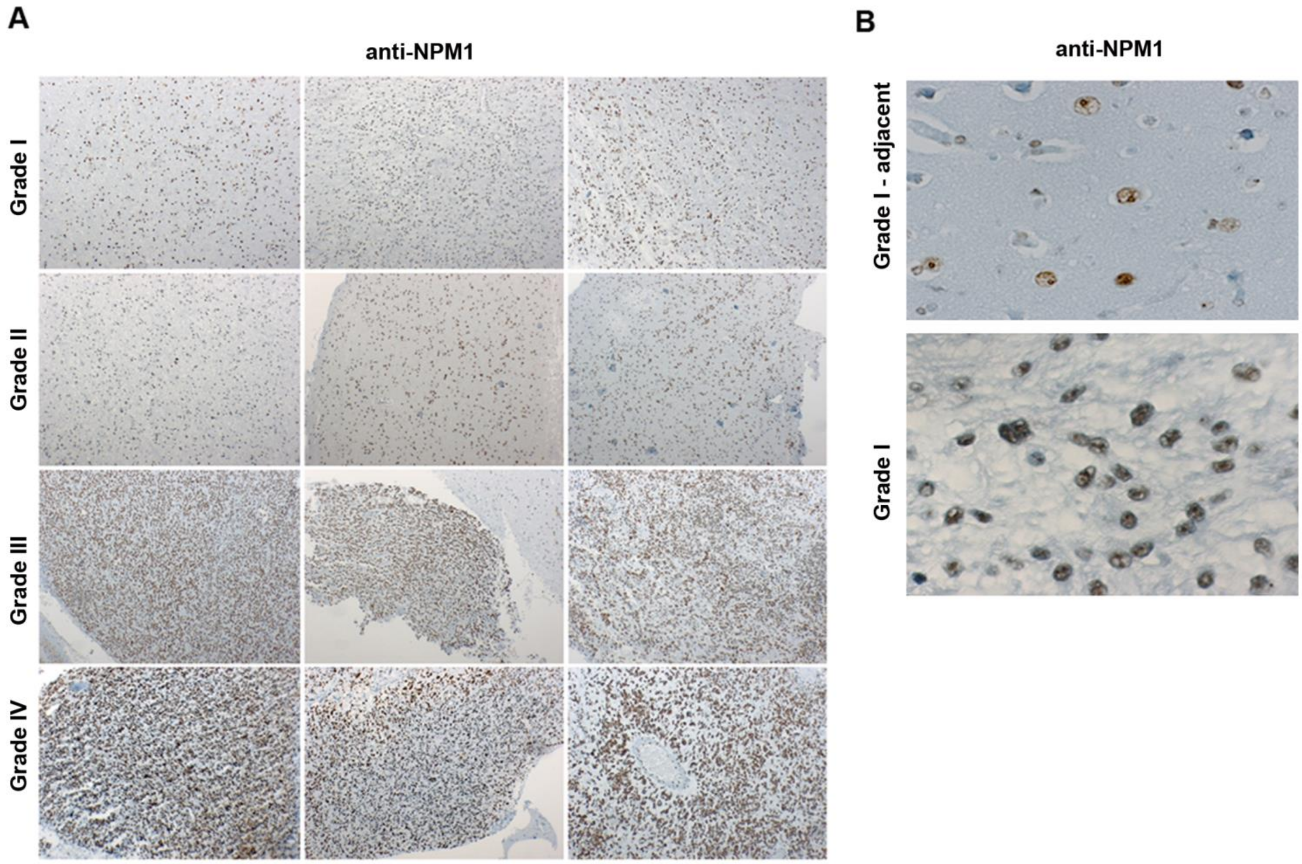

SUPPLEMENTARY FIGURE S1. Detection of NPM1 in astrocytic gliomas. A) Immunohistochemical staining of NPM1 in astrocytic glioma tumors of grades I, II, III and IV. Obj. 10x. B) Representative photomicrographs of NPM1 staining (brown) in adjacent near-normal area in a grade I tumor compared with a region from a grade I tumor with a higher density of cells. Obj. 40x.

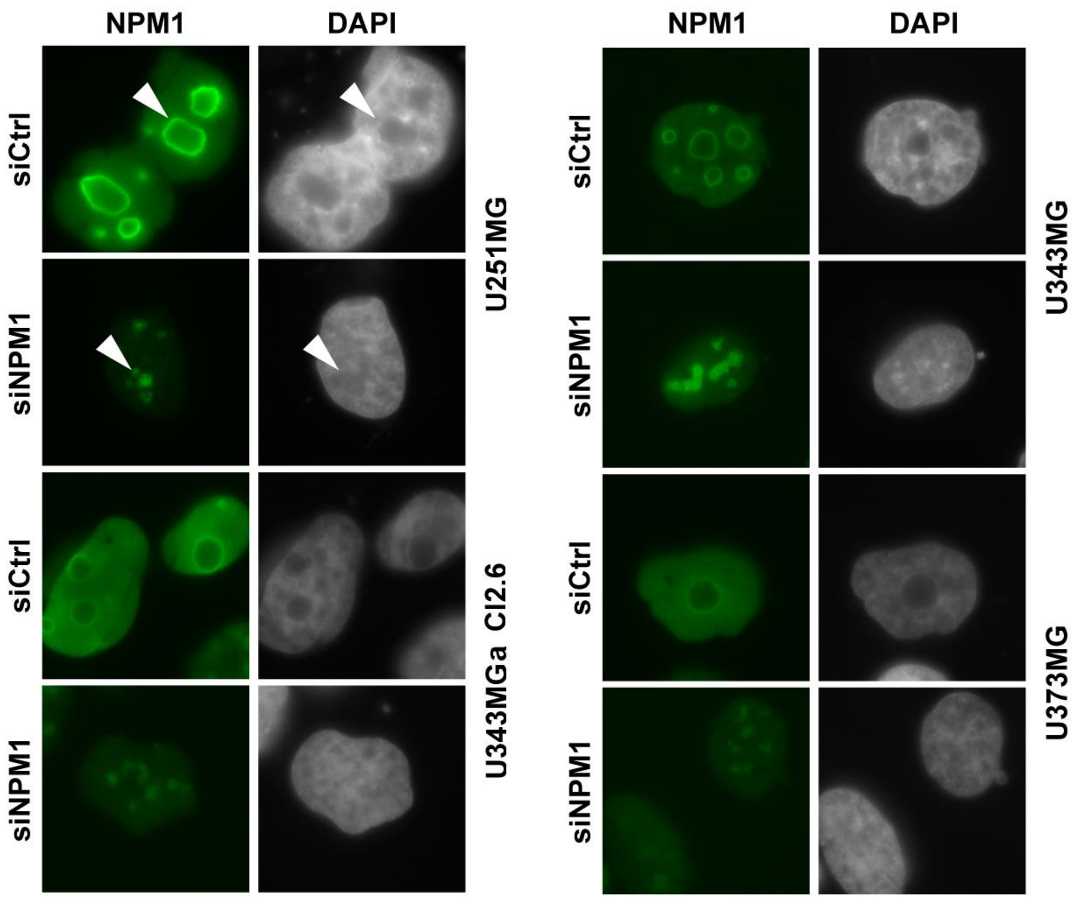

SUPPLEMENTARY FIGURE S2. NPM1 depletion alters the structure of nucleoli and surrounding chromatin in glioma cells. DAPI stainings of representative cell nuclei from glioma cell cultures with reduced levels of NPM1 indicate loss of the nucleolar structure. The darker DAPI-negative regions correspond to nucleoli. White arrowheads point at nucleoli in selected images. Cell cultures were transfected with siCtrl or siNPM1. Detection of NPM1 was made by immunofluorescence staining (green). Nuclei were counterstained with DAPI but visualized in grey.

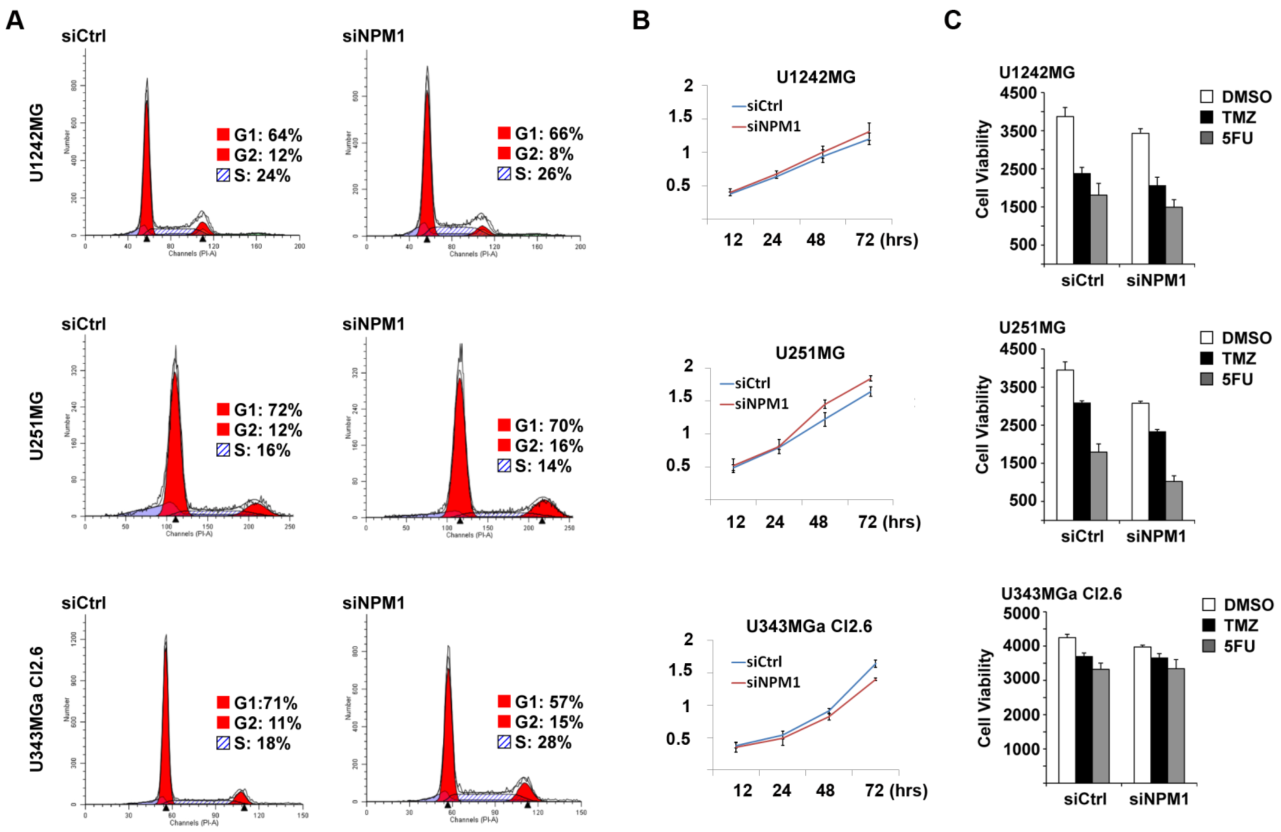

SUPPLEMENTARY FIGURE S3. Minor effects of NPM1 depletion on cell cycle distribution, cell proliferation, and TMZ and 5-FU induced cytotoxicity. A) Cell cycle analysis by FACS using glioma cell lines U1242MG, U251MG and U343MGa Cl2.6 treated with either siNPM1 and siCtrl for a 6 day period. Diagrams portray G1, G2, and S phase with percentage of cells in each phase for the separate glioma cell lines. B) Proliferation of glioma cells depleted of NPM1 was measured by the MTT assay. The cells had been depleted of NPM1 for 4 days prior to the start of the assay. Error bars represent the standard deviation of the experiment performed in triplicate. Y-axis represents absorbance in a relative scale. C) Cytotoxicity assay measurement (relative scale) of the viability of glioma cells treated with either TMZ (1mM), 5-FU (1mM) or DMSO (as control). Error bars represent the standard deviation in one representative experiment in triplicate.

A

Peptides shown in bold have been analysed by MS/MS sequencing

Start - End	Observed	Mr(expt)	Mr(calc)	Delta	Miss	Sequence
**37 - 49**	**1340.77**	**1339.76**	**1339.77**	**-8.00**	**1**	**R.KATGPPVSELITK.A (Ions score 55)**
38 - 49	1212.68	1211.67	1211.68	-4.00	0	K.ATGPPVSELITK.A
56 - 66	1171.67	1170.66	1170.67	-7.00	1	K.ERNGLSLAALK.K
58 - 67	1014.57	1013.57	1013.62	-55.00	1	R.NGLSLAALKK.A
**68 - 82**	**1564.79**	**1563.78**	**1563.76**	**10.00**	**1**	**K.ALAAGGYDVEKNNSR.I (Ions score 64)**
94 - 109	1538.82	1537.81	1537.81	-1.00	1	K.GTLVQTKGTGASGSFK.L

B

Linker histone H1

```
2. MSETAPAAPAAAPPAEKAPVKKKAAKKAGGTP---RKASGPPVSELITKAVAASKERSGV 57
3  MSETAPLAPTIPAPAEKTPVKKK-AKKAGATAGK-RKASGPPVSELITKAVAASKERSGV 58
4  MSETAPAAPAAPAPAEKTPVKKKARKSAGAAK---RKASGPPVSELITKAVAASKERSGV 57
5  MSETAPAETATPAPVEKSPAKKKATKKAAGAGAAKRKATGPPVSELITKAVAASKERNGL 60
1  MSETVPPAPAASAAPEKPLAGKKAKKPAKAAAASKKKPAGPSVSELIVQAASSSKERGGV 60
   ****.*  .: ...  **. .  **  * * .:    :*.:**.*****.:*.::****.*:

2. SLAALKKALAAAGYDVEKNNSRIKLGLKSLVSKGTLVQTKGTGASGSFKLNKKAASGEAK 117
3  SLAALKKALAAAGYDVEKNNSRIKLGLKSLVSKGTLVQTKGTGASGSFKLNKKAASGEGK 118
4  SLAALKKALAAAGYDVEKNNSRIKLGLKSLVSKGTLVQTKGTGASGSFKLNKKAASGEAK 117
5  SLAALKKALAAGGYDVEKNNSRIKLGLKSLVSKGTLVQTKGTGASGSFKLNKKAASGEAK 120
1  SLAALKKALAAAGYDVEKNNSRIKLGIKSLVSKGTLVQTKGTGASGSFKLNKKASSVETK 120
   ***********.**************:***************************:* * *

2. PKVKKAGGTKPKKPVGAAKKPKKAAGGATPKKSAKKTPKKAKKPAAATVTKKVAKSPKKA 177
3  PKAKKAGAAKPRKPAGAAKKPKKVAGAATPKKSIKKTPKKVKKPATAAGTKKVAKSAKKV 178
4  PKAKKAGAAKAKKPAGAAKKPKKATGAATPKKSAKKTPKKAKKPAAAAGAKK-AKSPKKA 176
5  PKAKKAGAAKAKKPAGAT--PKKAKKAAGAKKAVKKTPKKAKKP-AAAGVKKVAKSPKKA 177
1  PGASKV--ATKTKATGASKKLKKATGAS--KKSVK-TPKKAKKP---AATRKSSKNPKKP 172
   * ..*.  :.  *..**:   **.  .:  **: * ****.***   : .:* :*..**

2. KVA-KPKKAAKS--AAKAVK-----PKAAKP-----KVVKPKKAAPKKK 213
3  KTP-QPKKAAKSPAKAKAPK-----PKAAKPKSGKPKVTKAKKAAPKKK 221
4  KAA-KPKKAPKSPAKAKAVK-----PKAAKPKTAKPKAAKPKKAAAKKK 219
5  KAAAKPKKATKSPAKPKAVKPKAAKPKAAKPKAAKPKAAKAKKAAAKKK 226
1  KTV-KPKKVAKSPAKAKAVK-----PKAAKARVTKPKTAKPKKAAPKKK 215
   *.  :***..**   .** *     *****.     *..*.****.***
```

SUPPLEMENTARY FIGURE S4. Identification of H1.5 peptides in NPM1 nuclear complex co-IP. A) Peptides unique to linker histone H1.5 identified by MS/MS sequencing. B) Multiple sequence alignment of linker histone H1 form 1-5 according to Clustal W multiple sequence alignment software. Peptides unique to H1.5 are underlined in red in the alignment.

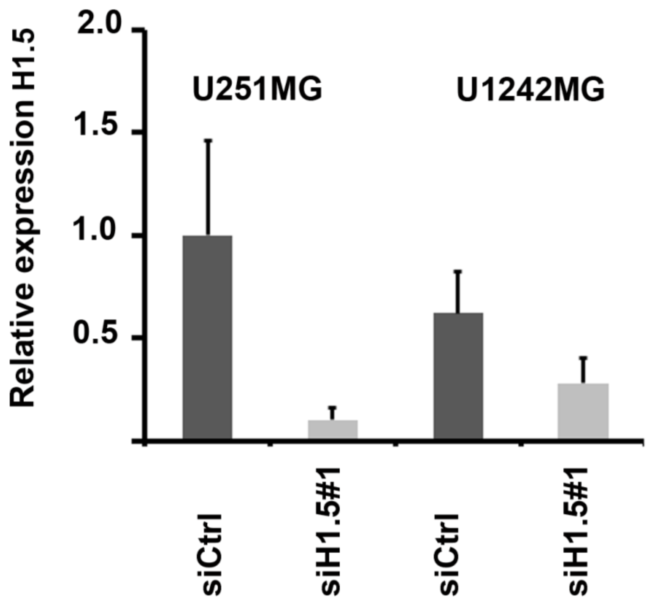

SUPPLEMENTARY FIGURE S5. Validation of H1.5 knockdown. Real-time quantitative reverse transcriptase-polymerase chain reaction was performed to monitor H.1.5 mRNA expression in U251MG and U1242MG cells that had been transfected with either H1.5 siRNA #1 or siCtrl for 24 hours. GAPDH was used as the internal standard reference. Error bars represent standard deviation of a triplicate measurement. This is a control experiment relevant to data shown in Fig. 6.
